# Synthesis of a 2-nitroimidazole derivative *N*-(4-[^18^F]fluorobenzyl)-2-(2-nitro-1*H*-imidazol-1-yl)-acetamide ([^18^ F]FBNA) as PET radiotracer for imaging tumor hypoxia

**DOI:** 10.1186/s41181-022-00165-0

**Published:** 2022-06-13

**Authors:** Arian Pérez Nario, Jenilee Woodfield, Sofia Nascimento dos Santos, Cody Bergman, Melinda Wuest, Yasniel Babí Araújo, André Luis Lapolli, Frederick G. West, Frank Wuest, Emerson Soares Bernardes

**Affiliations:** 1 Nuclear and Energy Research Institute (IPEN/CNEN - SP), São Paulo, SP CEP 05508-000 Brazil; 2 Department of Oncology, University of Alberta, Edmonton, AB T6G 2R7 Canada; 3 Department of Chemistry, University of Alberta, Edmonton, AB T6G 2G2 Canada

**Keywords:** *N*-(4-[^18^F]fluorobenzyl)-2-(2-nitro-1*H*-imidazol-1-yl)acetamide ([^18^F]FBNA), Hypoxia, Positron emission tomography (PET)

## Abstract

**Background:**

Tissue hypoxia is a pathological condition characterized by reducing oxygen supply. Hypoxia is a hallmark of tumor environment and is commonly observed in many solid tumors. Non-invasive imaging techniques like positron emission tomography (PET) are at the forefront of detecting and monitoring tissue hypoxia changes in vivo.

**Results:**

We have developed a novel ^18^F-labeled radiotracer for hypoxia PET imaging based on cytotoxic agent benznidazole. Radiotracer *N*-(4-[^18^F]fluorobenzyl)-2-(2-nitro-1H-imidazol-1-yl)acetamide ([^18^F]FBNA) was synthesized through acylation chemistry with readily available 4-[^18^F]fluorobenzyl amine. Radiotracer [^18^F]FBNA was obtained in good radiochemical yields (47.4 ± 5.3%) and high radiochemical purity (> 95%). The total synthesis time was 100 min, including HPLC purification and the molar activity was greater than 40 GBq/µmol. Radiotracer [^18^F]FBNA was stable in saline and mouse serum for 6 h. [^18^F]FBNA partition coefficient (log*P* = 1.05) was found to be more lipophilic than [^18^F]EF-5 (log*P* = 0.75), [^18^F]FMISO (log*P* = 0.4) and [^18^F]FAZA (log*P* =  − 0.4). In vitro studies showed that [^18^F]FBNA accumulates in gastric cancer cell lines AGS and MKN45 under hypoxic conditions.

**Conclusions:**

Hence, [^18^F]FBNA represents a novel and easy-to-prepare PET radioligand for imaging hypoxia.

## Background

Tissue hypoxia results from insufficient tissue oxygenation due to an imbalance between oxygen supply and oxygen consumption. Solid tumors often develop hypoxia due to the decreased delivery of oxygenated blood to meet the increased metabolic demands in rapidly proliferating and growing tumor cells. Currently, 60% of solid tumors in advanced stages contain hypoxic regions (Lv et al. 2017).

Hypoxia causes genomic changes by upregulating transcription factors like hypoxia-inducible factor 1 (HIF-1), resulting in tumor invasiveness and metastasis (Dhani et al. 2015). Tumor hypoxia is an obstacle to radiotherapy since the primary mechanism of tumor treatment with radiation is the creation of reactive oxygen species to cause deoxyribonucleic acid (DNA) damage, which is enhanced in the presence of oxygen. Under hypoxic conditions, cells cannot follow a sequence of deleterious reactions, which prevents tissue from being damaged, resulting in resistance to radiation and local disease recurrence (Barker et al. 2015). Consequently, patients with hypoxic tumors often have a poor prognosis and decreased overall survival rates.

Detection of hypoxia in tumors with radionuclides was first reported with ^14^C-labeled misonidazole autoradiography (Chapman 1979). From a PET imaging perspective, hypoxia imaging agents must readily and non-specifically enter cells, sample the intracellular milieu, and exit cells only in the presence of relevant oxygen concentrations.

Most clinically tested radiotracers for PET imaging of hypoxia represent ^18^F-labeled 2-nitro-imidazoles. Several excellent reviews have summarized the chemistry and application of PET radiotracers, emphasizing 2-nitroimidazoles for imaging hypoxia (Fleming et al. 2015; Wuest and Wuest 2013; Huang et al. 2021). Nitroimidazoles enter cells by passive diffusion followed by their reduction to nitro radical anions, which are further reduced to nitroso- and hydroxylamine compounds in the presence of hypoxic conditions leading to intracellular trapping (Mees et al. 2009).

The most extensively used hypoxia imaging radiotracer for PET in the clinic is ^18^F-labeled 2- nitroimidazole [^18^F]-Fluoromisonidazole ([^18^F]FMISO) (Jerabek et al. 1986). However, a more general clinical use of [^18^F]FMISO is hampered due to its slow tumor-specific accumulation, slow clearance of unbound tracer from normoxic tissue, and high radioactivity concentration in the digestive tract. A sufficient lipophilicity window of a hypoxia imaging agent is crucial to enter cells and allow uniform tissue distribution and sufficiently lipophilic; however, it is still not clear the opening width of such window and/or if there are any other factors capable of counterbalancing lipophilicity to reduce image noise and improve image quality.

**Fig. 1:**
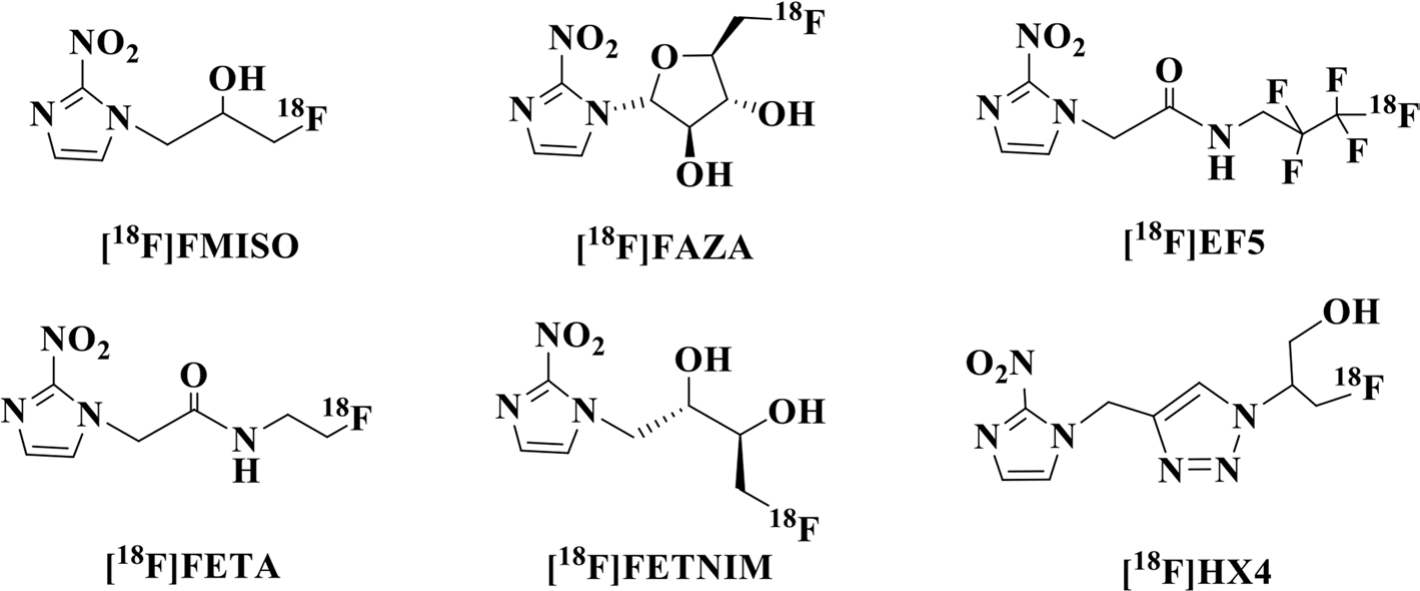
Chemical structures of selected ^18^F-labeled 2-nitroimidazoles used for PET imaging of tissue hypoxia

Thus, several next-generation hypoxia imaging PET radioligands have been developed and tested to overcome these limitations (Fleming et al. 2015). Prominent examples include ^18^F-labeled [^18^F]fluoroazomycin arabinoside ([^18^F]FAZA) (Postema et al. 2009), [^18^F]fluoroerythronitroimidazole ([^18^F]FETNIM) (Grönroos et al. 2001), [^18^F]fluoroetanidazole ([^18^F]FETA) (Barthel et al. 2004) and [^18^F]labeled flortanidazole ([^18^F]HX4) (Wack et al. 2015), all of them much less lipophilic than [^18^F]FMISO, and the highly lipophilic 2-(2-nitro-1H-imidazol-1-yl)-*N*-(2,2,3,3,3-[^18^F]penta-fluoropropyl)-acetamide ([^18^F]EF5) (Dolbier et al. 2001). A selection of representative ^18^F-labeled 2-nitro-imidazoles as radiotracers for PET imaging of hypoxia is given in Fig. 1.

Examples of ^18^F-labeled 2-nitroimidazoles shown in Fig. 1 involve radiotracers with ^18^F attached to a sp^3^ carbon. To the best of our knowledge and our surprise, only Raid et al. in 2010 reported ^18^F-aryl bond 2-nitroimidazoles for PET imaging of hypoxia (Raid et al. 2010).

**Fig. 2:**
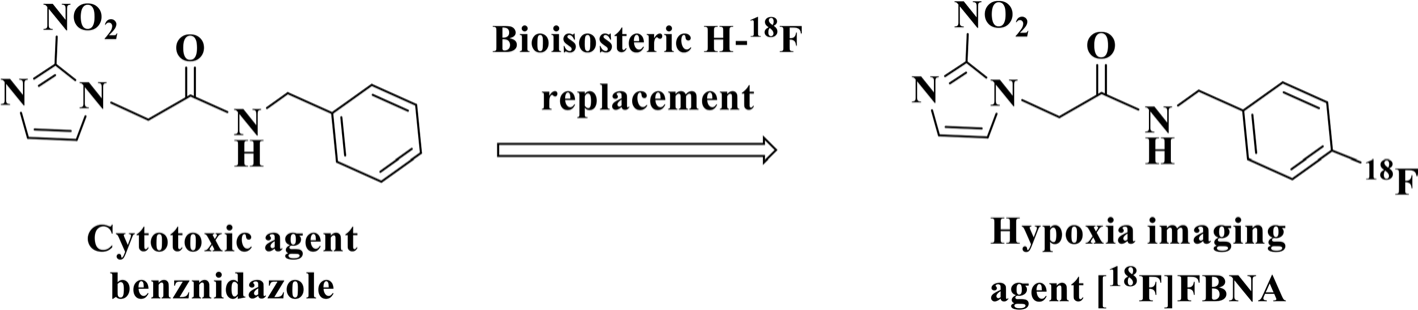
Chemical structures of benznidazole and [^18^F]FBNA

Benznidazole is a 2-nitroimidazole compound that becomes an active cytotoxic agent upon biological reduction mediated by nitroreductases (Li et al. 2016). The drug is currently used to treat Chagas disease caused by the parasitic protozoan *Trypanosoma cruzi*. Benznidazole also demonstrated cell killing capacity of hypoxic tumor cells. Bioisosteric replacement of hydrogen in the para position of benznidazole with F would result in fluorinated version of the compound suitable for radiolabeling with ^18^F to give benznidazole derivative, *N*-(4-[^18^F]fluorobenzyl)-2-(2-nitro-1*H*-imidazol-1-yl) acetamide ([^18^F]FBNA), for PET imaging of hypoxia (Fig. 2). The idea that FBNA could be used as a hypoxic imaging marker for PET by labeling its fluorine with ^18^F was first proposed in 1992 (Garg et al. 1992), but no further synthesis with ^18^F nor biological studies were subsequently published.

In this work, we have developed a new nitroimidazole derivative, *N*-(4-[^18^F]-fluorobenzyl)-2-(2-nitro-1*H*-imidazol-1-yl) acetamide ([^18^F]FBNA), an ^18^F-labeled analogue of cytotoxic compound benznidazole. Reported herein are the synthesis, purification and chemical characterization of radiotracer [^18^F]FBNA. Radiotracer [^18^F]FBNA was found to be more lipophilic than [^18^F]EF-5, [^18^F]FMISO and [^18^F]FAZA, and it was quickly trapped in tumor cells under hypoxic conditions.

## Results

### Design concept and synthesis of the labeling precursor and reference compound

**Scheme 1:**
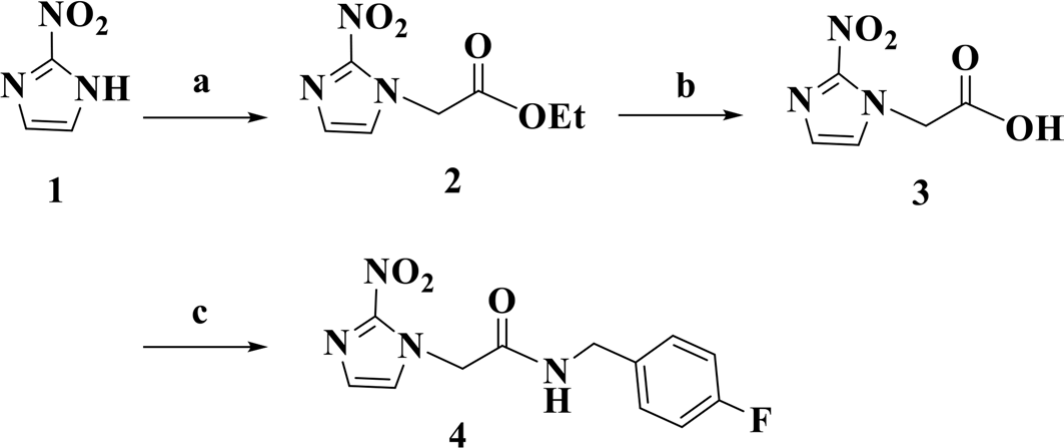
Synthesis of the labeling precursor **3** and reference compound **4**. Reagents and conditions: (a) CH_3_CO_2_CH_2_CH_2_Br, K_2_CO_3_, CH_3_CN, rt, 21 h; (b) 4.0 N NaOH, H_2_O/MeOH, rt, 3 h; (c) 4-Fluorobenzylamine, trifluoroacetic acid anhydride, THF, 0 °C, 1 h

Reference compound *N*-(4-fluorobenzyl)-2-(2-nitro-1H-imidazol-1-yl) acetamide (FBNA) was prepared by a three-step synthesis route, as shown in Scheme 1.

The synthesis of 2-nitroimidazole acetic acid **3** involved a nucleophilic addition reaction between ethyl-2-bromoacetate and 2-nitroimidazole **1** in the presence of the K_2_CO_3_, followed by basic hydrolysis of intermediate ethyl ester **2** using 4.0 N NaOH. The reaction resulted in the formation of compound **3** as a yellow solid in 69.5% yield for the two steps. Proton nuclear magnetic resonance (^1^H-NMR) and high-resolution mass spectrometry analysis confirmed the chemical structure of compound **3**. Reference compound **4** was obtained through acylation reaction of in situ generated mixed anhydride of carboxylic acid **3** in the presence of 4-fluorobenzyl amine. Compound 4, was isolated in 41.3% yield with a total synthesis time of 5 h.

### Radiosynthesis of [^18^F]FBNA

**Scheme 2:**
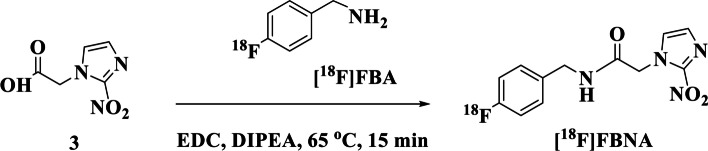
Radiosynthesis of [^18^F]FBNA from its precursor, 2-nitroimidazole acetic acid (**3**)

The radiosynthesis of radiotracer [^18^F]FBNA is shown in Scheme 2.

The fully automated radiosynthesis of 4-[^18^F]fluorobenzylamine ([^18^F]FBA) was accomplished according to a method recently published by Way and Wuest (2013). Briefly, to azeotropically dried [^18^F]fluoride/K_222_, 4-cyano-*N,N,N*-trimethylanilinium trifluoro-methansulfonate (10 mg) in CH_3_CN (1 mL) was reacted for 15 min at 90 °C.

After purification using a Phenomenex Strata C-18U cartridge, [^18^F]FBA was directly eluted with THF (2.0 mL) into reactor 2 of a GE TRACERlab™ FX automated synthesis unit and diluted with water (12 mL). The reaction mixture was passed through the activated reducing cartridge to reduce 4-[^18^F]fluorobenzonitrile ([^18^F]FBN) to [^18^F]FBA. The radiochemical yield of [^18^F]FBA was 69% ± 9% (n = 7), and the radiochemical purity, as determined by radio-Thin Layer Chromatography (radio-TLC) was 84% ± 15%. In a typical experiment, 21.6 GBq of cyclotron-produced [^18^F]fluoride could be converted into 9.3 GBq of [^18^F]FBA within a total synthesis time of 90 min.

**Fig. 3:**
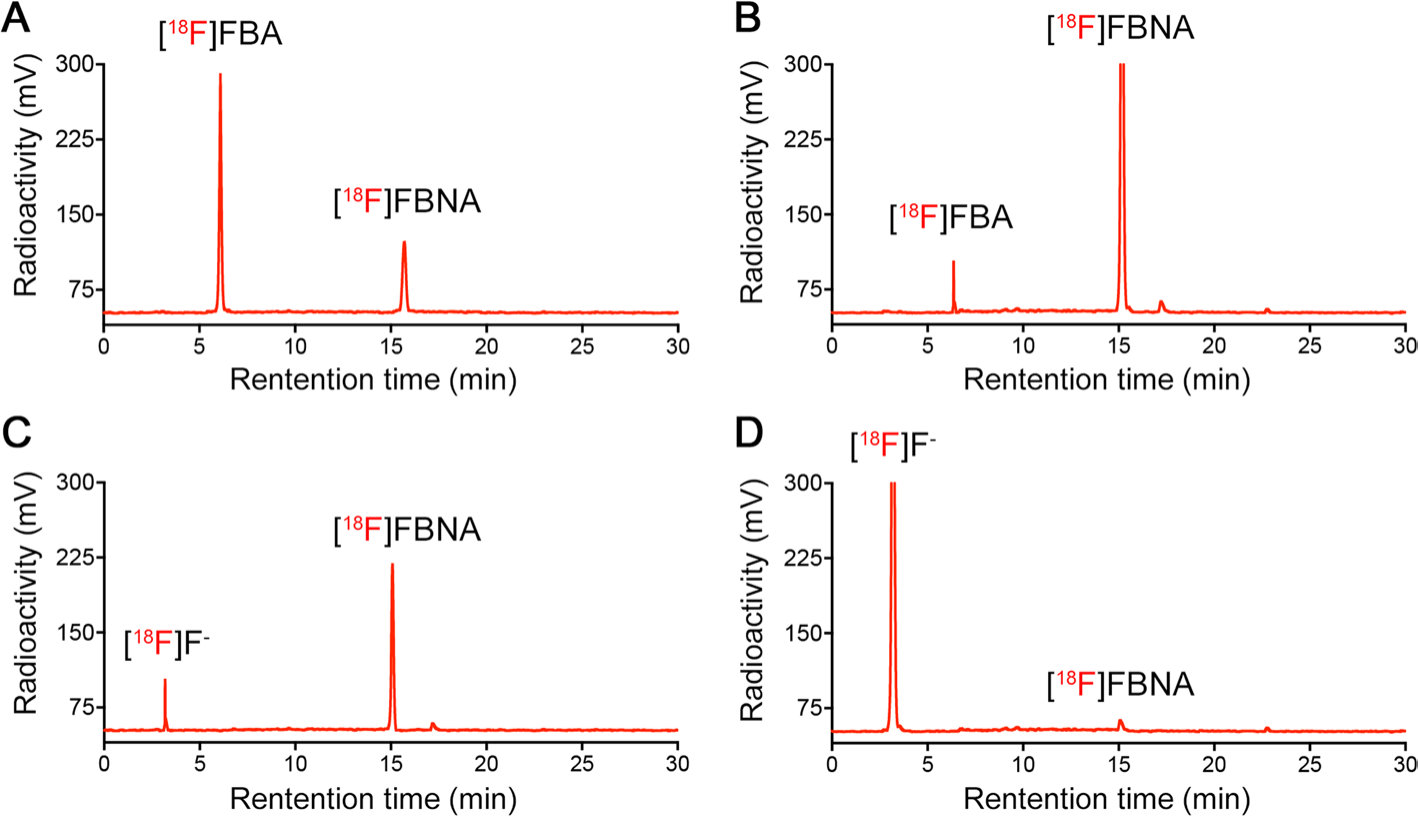
Radio-HPLC traces of the reaction ([^18^F]FBNA: t_*R*_. = 15.1 ± 0.3 min) performed at room temperature (**A**), 65 °C (**B**), 85 °C (**C**) and 100 °C (**D**)

The radiosynthesis of [^18^F]FBNA was accomplished via acylation reaction of activated carboxylic acid group in compound **3** with [^18^F]FBA. We studied the effect of temperature on the radiolabeling yield using radio-High Performance Liquid Chromatography (radio-HPLC) analysis (Fig. 3). At room temperature, the radiochemical yield of [^18^F]FBNA was 42%. We observed the presence of large quantities of unreacted [^18^F]FBA, indicating that the reaction was not fully completed (Fig. 3A). At 65 °C, [^18^F]FBNA radiochemical yield increased to 85% (Fig. 3B). A further increase in temperature to 85 °C led to complete consumption of [^18^F]FBA to form radiotracer [^18^F]FBNA while starting radiodefluorination was noticeable (Fig. 3C). When the temperature was increased to 100 °C, more than 90% of the product was degraded, as indicated by forming a large [^18^F]fluoride peak (Fig. 3D).

**Fig. 4:**
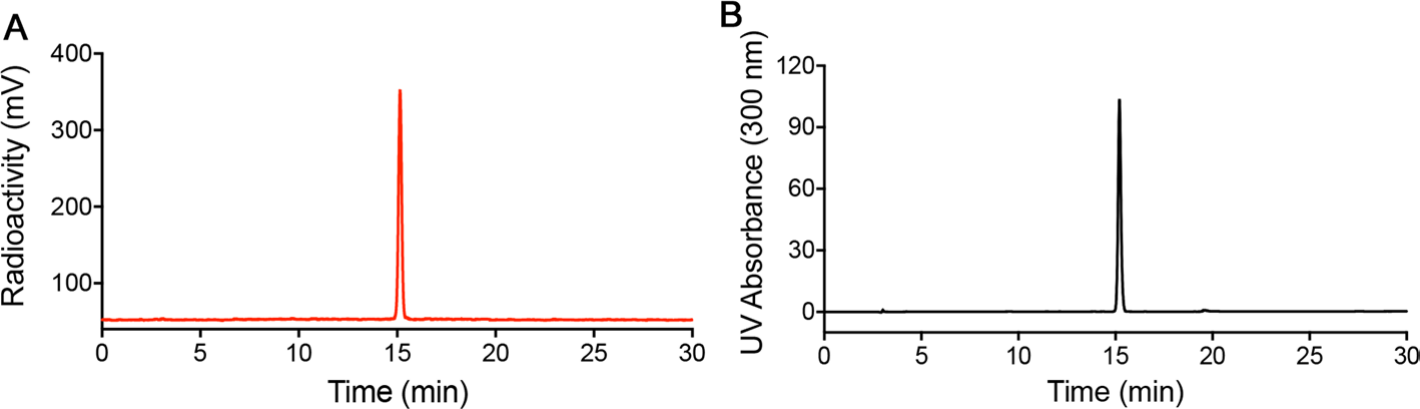
HPLC traces of [^18^F]FBNA (left) ([^18^F]FBNA: t_*R*_. = 15.1 ± 0.3 min) and reference compound FBNA (right) (FBNA: t_*R*_. = 15.0 ± 0.3 min). UV detector was installed ahead of radioactivity detector

Radioligand [^18^F]FBNA was purified by HPLC and identified by co-injection of reference compound FBNA (Fig. 4A, B). The pH of the final solution in 300 µL saline solution was 7.2. The molar activity of [^18^F]FBNA was at least 40 GBq/µmol, and the decay-corrected radiochemical yield was 47.4% ± 5.4% (n = 5), corrected based on the decay of [^18^F]fluoride. The radiochemical purity was determined by radio-HPLC and exceeded 95%. In a typical experiment, starting from 3.9 GBq of [^18^F]fluoride, 0.9 GBq of [^18^F]FBNA was obtained within 100 min, including HPLC purification.

Hypoxia imaging agents [^18^F]FAZA and [^18^F]FMISO were also prepared using a fully automated synthesis procedure for comparative studies with [^18^F]FBNA. The radiochemical yield of [^18^F]FAZA and [^18^F]FMISO was 24.8% ± 7.4% and 58.1% ± 17.9% (n = 8), respectively. The radiochemical purity as determined by radio-HPLC exceeded 99%, and the molar activity of [^18^F]FMISO and [^18^F]FAZA was > 40 GBq/μmol.

### In vitro stability studies

**Fig. 5:**
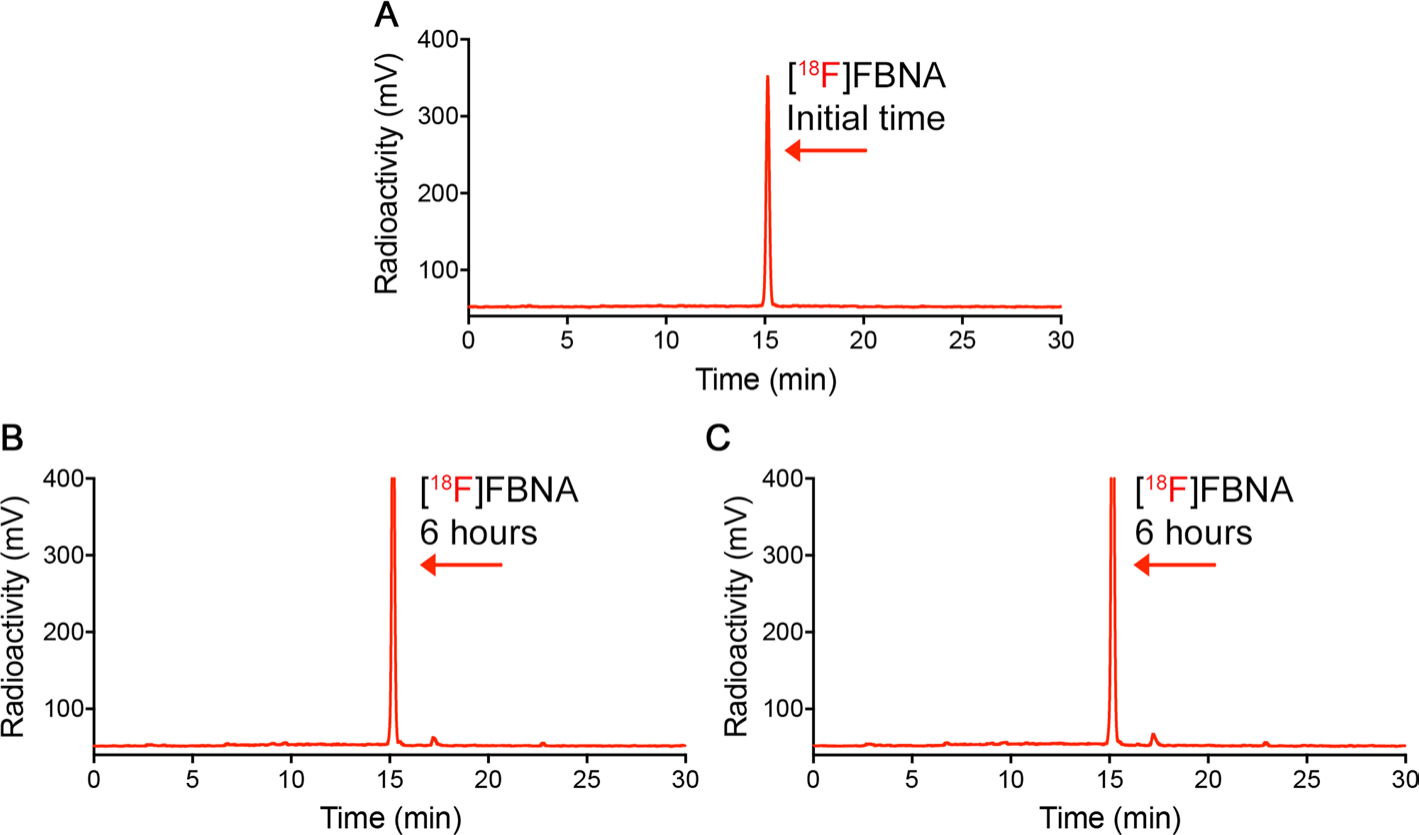
Radio-HPLC traces obtained after the incubation of [^18^F]FBNA (t_*R*_: 15.2 min) at (**A**) the initial time (t = 0 min), (**B**) physiological saline solution (NaCl 0.9%) after 6 h or (**C**) mouse serum after 6 h

The stability assessment of radiolabeled compounds is crucial to determine the suitability of a radiotracer for in vivo applications. In this work, the stability of radiotracer [^18^F]FBNA was evaluated in a physiological saline solution (NaCl 0.9%) and mouse serum over a time course of 6 h. We observed that at the initial time, [^18^F]FBNA displayed a single peak upon radio-HPLC analysis at 15.2 min (Fig. 5A). Radiotracer [^18^F]FBNA remained stable in saline solution at room temperature (Fig. 5B) and in mouse serum at 37 °C (Fig. 5C) over 6 h.

### Determination of the partition coefficient (Log *P*)

The partition coefficient determination for [^18^F]FBNA, [^18^F]FMISO, [^18^F]FAZA was conducted according to Wilson et al. 2001. It was found that [^18^F]FBNA had a log*P* = 1.05 ± 0,04 which was more lipophilic than [^18^F]FMISO (log*P* = 0.36) and [^18^F]FAZA (log*P* =  − 0.43) (Table 1). The Log*P* values obtained for [^18^F]FMISO and [^18^F]FAZA were similar to those reported by other authors (Fleming et al, 2015).

**Table 1:** Partition coefficient of [^18^F]FBNA. Compared with [^18^F]FAZA and [^18^F]FMISO

Radiotracer	Partition coefficient (*P*)	Partition coefficient (log *P*)
[^18^F]FBNA	11.21 ± 0.10	1.05 ± 0.04
[^18^F]FAZA	0.37 ± 0.08	− 0.43 ± 0.03
[^18^F]FMISO	2.29 ± 0.12	0.36 ± 0.03

### Cellular uptake studies

**Fig. 6:**
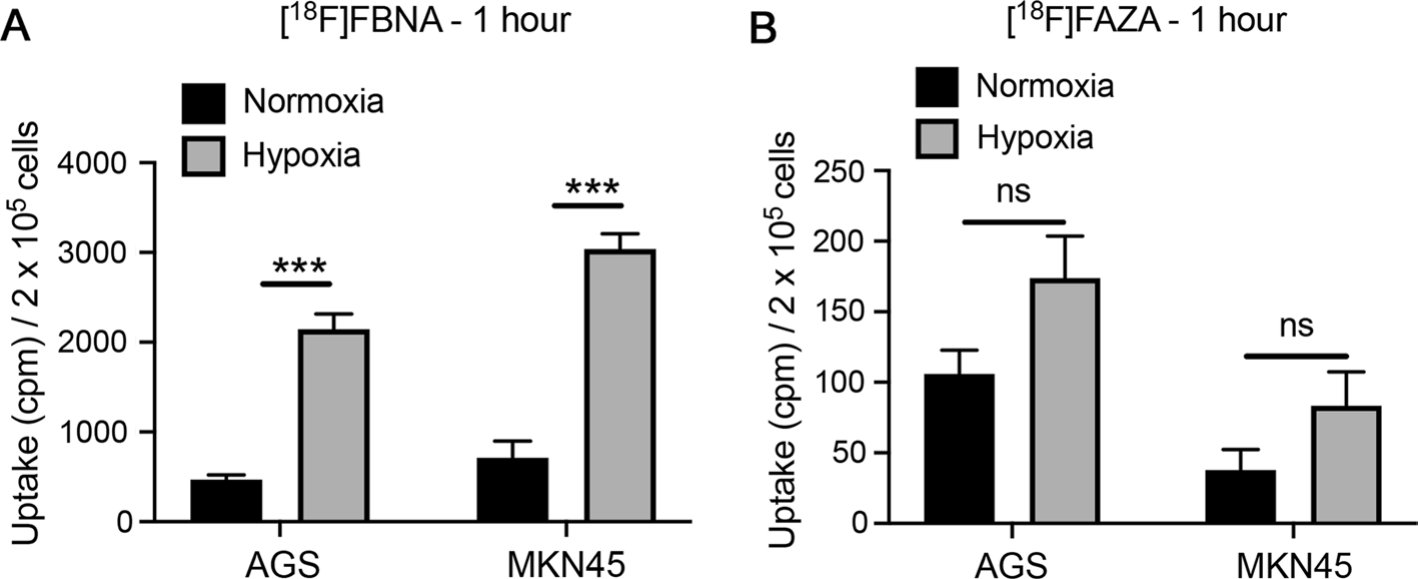
In vitro uptake assay. AGS and MKN45 cells were seeded in a 6-well plate and cultured for 48 h under normoxic (21% O_2_) or hypoxic (1% of O_2_) for 48 h. After this period, 0.37 MBq of (**A**) [^18^F]FBNA or (**B**) [^18^F]FAZA was incubated for 1 h. Bars show the mean ± standard deviation (SD) of the uptake of the radiotracer (cpm)/2 × 10^5^ cells of three independent experiments (n = 3). Data were analyzed by one unpaired *t*-test (multiple *t* tests); *p < 0.5, ****p < 0.0001 (Holm-Sidak method)

Finally, we evaluated the ability of radiotracer [^18^F]FBNA to be retained in tumor cells under hypoxic conditions. [^18^F]FAZA was used for comparison. Briefly, the gastric cancer cell lines AGS and MKN45 were cultured under normoxic (21% of O_2_) or hypoxic conditions (1% of O_2_) for 48 h before the addition of radiotracers [^18^F]FBNA or [^18^F]FAZA and incubation for 1 h. The uptake of radiotracer [^18^F]FBNA (Fig. 6A) was significantly higher under hypoxic than under normoxic conditions (4.5 times for AGS and 4.2 times for MKN45 cells). On the other hand, 1 h of incubation with the less lipophilic radiotracer [^18^F]FAZA showed an increased uptake under hypoxic conditions, still to a lower extend, 1.6 times for AGS and 2.1 times for MKN45 (Fig. 6B). The data demonstrate that radiotracer [^18^F]FBNA displays significantly higher cellular uptake compared to radiotracer [^18^F]FAZA.

## Discussion

In this study, we show the development of a novel highly lipophilic PET radiopharmaceutical, [^18^F]FBNA. The novel radiotracer is easy to prepare, displays a high affinity for hypoxic tumor cells and may represent a further step towards a better understanding of the characteristics of an ideal hypoxia PET imaging agent.

Among the several radiopharmaceuticals for PET imaging of tumor hypoxia, [^18^F]-FMISO is the most extensively studied and the first 2-Nitroimidazole-derived PET radiotracers used clinically (Grierson et al. 1989). So far, [^18^F]FMISO has been investigated in several clinical trials for different purposes such as: (1) radiotherapeutic planning based on hypoxic subvolumes in tumors (Hendrickson et al. 2011; Lee and Scott 2007); (2) prognostic images to predict therapeutic outcome based on hypoxic fractions (Spence et al. 2008; Grkovski et al. 2017) (3) hypoxia volume (HV), degree (maximum ratio [Rmax]) defined from [^18^F]FMISO uptake rates (Randy 2009) (4) correlation with progression free survival, overall survival (Askoxylakis et al. 2012) and (5) Maximum Standardized Uptake Value (SUVmax) in tumor and normal tissue. However, because of its slow accumulation in hypoxic tissue and slow elimination, it often results in low contrast diagnostic images. In addition, its predominantly hepatic elimination results in a high dose of radiation released to the liver (Piert 2009).

**Fig. 7:**
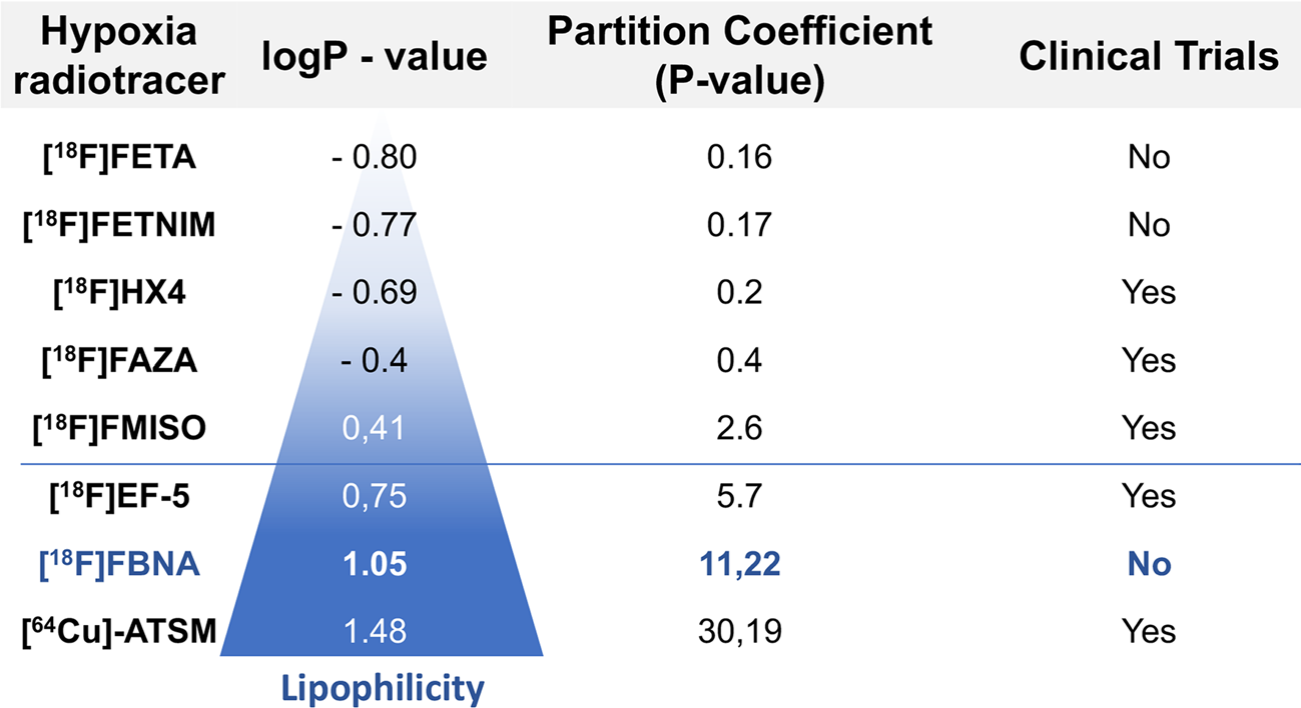
Characteristic of hypoxic PET tracers under development and number of clinical trials

To overcome the disadvantages of [^18^F]FMISO, a second and third generation of more water-soluble (hydrophilic) hypoxia PET radiopharmaceuticals have been developed (Fig. 7); among them, [^18^F]FAZA (LogP − 0.4) [^18^F]FETNIM (LogP − 0.77) and [^18^F]FETA (LogP − 0.80) (second generation), and [^18^F]HX4 (LogP − 0.69) (third generation), have showed great results in preclinical and clinical studies with a selective accumulation in hypoxic tumor cells and improved tumor-to-background ratios when compared to [^18^F]FMISO (Sorger et al. 2003; Piert et al. 2005; Yang et al. 1995; Peeters et al. 2015 and Wack et al. 2015). However, the reproducibility of PET data for [^18^F]FAZA is still very variable and evidence of its superiority in relation to [^18^F]FMISO for image-guided radiotherapy is still limited. In addition, it is essential to wait a period of 3 h after the injection of the radiopharmaceutical to obtain optimal images in preclinical models and in patients (Postema et al. 2009). [^18^F]-FAZA has nowadays 14 ongoing clinical trials. [^18^F]HX4 on the other hand, has shown an enhanced renal clearance. Still, bladder voiding needs to be ensured to prevent an increased bladder wall dosimetry (Doss et al. 2010) At least 10 clinical trials have studied the clinical relevance of [^18^F]HX4.

Although much has been said about high relative lipophilicity of [^18^F]FMISO, which leads in a significant low uptake, a highly lipophilic fluorinated radiopharmaceuticals, [^18^F]EF-5 (LogP 0.75), has also been investigated as promising alternatives to [^18^F]FMISO (Koch et al. 1995; Dolbier et al. 2001). [^18^F]EF-5 was first reported by Dolbier et al. (2001) as well suited hypoxic PET tracer. Indeed, [^18^F]EF-5 was shown to be a promising agent in providing early responses to treatment and predictions of results in chemoradiotherapy and radiotherapy in preclinical models (Chitneni et al. 2011; Ali et al. 2015). Clinical studies with [^18^F]EF-5 imaging have demonstrated a clinically acceptable biodistribution, dosimetric and imaging profile in patients with head and neck cancer (Komar et al. 2014; Silvoniemi et al, 2018; Wright et al, 2021) and cervical cancer (Narva et al, 2021). Further studies evaluating the clinical use of [^18^F]EF-5 in head and neck cancer (NCT05246475) and ovarian cancer (NCT04001023) are still ongoing.

In the present work we have developed a novel highly lipophilic 2-nitroimidazole-derivative PET tracer, [^18^F]FBNA with a LogP 1.05, which is much easier to prepare in comparison to [^18^F]EF-5 (Koch et al. 2010; Challapalli et al. 2017).

Considering non ^18^F-labeled 2-nitro-imidazoles representatives, [^64^Cu]ATSM (LogP 1.48) is more lipophilic than [^18^F]FBNA and has been evaluated in 5 clinical trials (NCT04875871 and NCT03951337 are currently ongoing), as a promising alternative to 2-nitroimidazole compounds for hypoxia PET imaging. Indeed, [^64^Cu]ATSM is a highly membrane permeable complex with a rapid blood clearance and high intertissue and intratumoral contrast, features conferred by its lipophilic characteristics (Hansen et al. 2012). However, [^64^Cu]ATSM is more than a hypoxic tracer and can accumulate in tumors depending on the redox potential and reactive oxygen species of cells, and thus, is an indirect marker for hypoxic PET imaging (Colombié et al. 2015; Vavere and Lewis 2008). Therefore, despite all the excitement about [^64^Cu]ATSM, its mechanism of action is still unclear, and more studies are needed to prove its diagnostic utility.

Altogether, our results demonstrate the preparation of a potential clinically relevant novel radiopharmaceutical, [^18^F]FBNA, as non-invasive hypoxia biomarker for PET imaging. In addition of being a novel highly lipophilic 2-nitroimidazole-derivative, [^18^F]FBNA is a fluorinated version of Benznidazole, which is considered first-line treatment of Chagas disease, a neglected tropical diseases caused by *Trypanosoma cruzi* (Bern et al. 2007). Therefore, we are currently performing in vivo studies not only to demonstrate the suitability of [^18^F]FBNA as the next hypoxic imaging agent but also as a potential tracer to detect *Trypanosoma cruzi* in infected patients.

## Conclusion

In this study, we established a robust and reproducible radiosynthesis of hypoxia imaging agent [^18^F]FBNA. Radiotracer [^18^F]FBNA represents a novel hypoxia imaging agent for PET containing a 4-[^18^F]fluorobenzyl group as a bioisosteric group of the benzyl group in parent compound benznidazole, a cytotoxic agents based on a 2-nitroimidazole motif. Radiotracer [^18^F]FBNA displayed a favorable cellular uptake profile in cancer cells under hypoxic conditions. We used readily available 4-[^18^F]fluorobenzyl amine ([^18^F]FBA) as key building block for the radiosynthesis of radiotracer [^18^F]FBNA. However, the current method is unsuitable for large-scale production since the acylation step for amide bond formation requires a manual procedure. Our group is working on a fully automated radiosynthesis for the large-scale preparation of [^18^F]FBNA, thus enabling the safe and straightforward synthesis of multiple GBq doses of the radiotracer for preclinical and eventual clinical applications. We are currently performing in vivo studies with radiotracer [^18^F]FBNA in tumor-bearing mice to investigate the biodistribution and pharmacokinetic profile of radiotracer [^18^F]FBNA in comparison to established hypoxia imaging agents [^18^F]FMISO and [^18^F]FAZA.

## Methods

### General

The precursor used for the preparation of [^18^F]FMISO, 1-(2′-nitro-1′-imidazolyl)- 2-*O*-tetra- hydropyranyl-3-*O*-toluenesulfonylpropane-diol (NITTP) and [^18^F]FAZA, 1-(2,3-di-*O*-acetyl-5-*O*- tosyl-α-D-arabinofuranosyl)-2-nitroimidazole, as well as the reference compounds for FMISO and FAZA were purchased from ABX advanced biochemical compounds (Radeberg, Germany). All reagents and solvents were of analytical reagent grade and were used without further purification unless otherwise specified. (TLC) was performed on precoated plates of silica gel 60 F254 (Merck, Darmstadt, Germany). ^1^H-NMR and Carbon-13 nuclear magnetic resonance (^13^C-NMR) spectra were recorded on an Agilent/Varian Inova two-channel 400 MHz spectrometer, an Agilent/Varian Inova four-channel 500 MHz spectrometer and an Agilent/Varian VNMRS three-channel 600 MHz spectrometer. Chemical shifts are given in ppm referenced to internal standards (s = singlet, singlet, d = doublet). High-resolution mass spectrometry (HR-MS) was carried out on an Agilent Technologies 6220. Solid-phase extraction cartridges (Oasis HLB (3 cm^3^) cartridge, Sep-Pak light QMA cartridge, neutral Al_2_O_3_ cartridge, and Sep-pak C-18 cartridge) were obtained from Waters (Milford, MA, USA). Radiosynthesis of 4-[^18^F]fluorobenzylamine [^18^F]FBA, [^18^F]FAZA, and [^18^F]FMISO were performed on a GE TRACERlab™ FX (General Electric Company, Fairfield, Connecticut, United States). The synthesis unit was installed in a hot cell allowing for safe handling of a large amount of activity to be utilized.

High-performance liquid chromatography (HPLC) purification and analysis of the radiolabeled products were performed using the Phenomenex LUNA® C18(2) column (100 Å, 250 × 10 mm, 10 μm) and Agilent C18, 4.6 mm × 250 mm, 5 μ, 100 Å respectively and the HPLC system was from Gilson 321 pump, 171 diode array detector, Berthold Technologies Herm LC. Experiments involving serum from live animals were approved by the Animal Ethics Committee of the Nuclear and Energy Research Institute (IPEN-CNEN/SP), São Paulo, Brazil. (Protocol number: 54/15/CEUA-IPEN/USP).

### Chemical syntheses

#### Synthesis of 2-nitroimidazole acetic acid 3

2-Bromoethyl acetate (216 µL, 1.32 mmol) was added to a solution of 2-nitroimidazole (200 mg, 1.77 mmol) and potassium carbonate (741 mg, 0.69 mmol) in dry acetonitrile (10 mL). The mixture was reacted under constant stirring for 21 h at room temperature, and the resulting precipitate was filtered of and washed with acetone (3**×**). Evaporation of the filtrate under reduced pressure yielded a yellow oil (310 mg; 1.56 mmol). Analytical TLC revealed a pure product; R_*f*_ = 0.4, EtOA/hexane, 7:3). The purified nitroimidazole ethyl acetate **2** (310 mg, 1.56 mmol) was added to a solution of 4 N NaOH (377 µL, 1.56 mmol), water (2 mL), and MeOH (2 mL). The resulting solution was stirred at room temperature for 3 h until no ester derivative was present (TLC analysis: CH_2_Cl_2_/MeOH, 9:1; R_*f*_ = 0.2). Cation-exchange resin (H + , Bio-Rad, 4 g), which had been conditioned by washing with 1.0 N H_2_SO_4_ (10 mL) and distilled water, was used to acidify the solution.

Filtration and drying of the filtrate yielded a dark yellow solid. Column chromatography of the crude product on silica gel [eluent, CH_2_Cl_2_/CH_3_OH (95:5)] yielded a yellow solid of compound **3** (201 mg, 97%): ^1^H-NMR (400 MHz, CD_3_OD) δ: 11.91 (s, 1 H), 7.39 (s, 1 H), 7.11 (s, 1 H), 3.31 (s, 2 H), HR-MS (ESI) calculated for C_5_H_5_N_3_O_4_H^−^[M + H]^−^: 170,0207; found, 170,0210.

#### Synthesis of *N*-(4-fluorobenzyl)-2-(2-nitro-1*H*-imidazol-1-yl)acetamide 4 (FBNA)

A mixture of acetic acid **3** (41 mg, 0.238 mmol) and trifluoroacetic anhydride (31.8 µL, 0.24 mmol) in 2 mL of anhydrous THF was mixed at 0 °C for 30 min, then 4-fluorobenzylamine (136.1 uL, 0.24 mmol) was added. The mixture was stirred at room temperature for an additional 30 min. Once the reaction was complete as monitored by TLC (CH_2_Cl_2_/MeOH, 9:1; R_*f*_ = 0.4), the filtrate was concentrated, and the crude product was purified by column chromatography (CH_2_Cl_2_/MeOH, 9:1 *v:v*) providing reference compound **4**. Yield 41.3% (20.1 mg, 0.07 mmol); Rf = 0.3 (DCM: MeOH 9: 1 v: v). ^1^H-NMR. δ_H_ (400 MHz, CD_3_OD) 8.77 (s, 1 H), 7.58 (dd, J = 1.1 Hz, 2.3 Hz, 1H), 7.25 (ddd, J = 2.6 Hz, 5.1 Hz, 8.0 Hz, 2H,), 7.10 (m, 3 H), 5.19 (s, 2H), 4.23 (d, J = 5.1 Hz, 2H). ^13^C-NMR. δc (CD_3_OD, 150 MHz), 166.23, 162.51, 160.91, 135.49, 129.70, 129.29, 127.99, 115.58, 52.04, 42.07. HR-MS (ESI) calculated for C_12_H_11_FN_4_O_3_[M + H]^+^: 279.0815, found: 279.0886.

### Radiochemistry

#### Production of no-carrier-added (n.c.a.) [^18^F]fluoride

No-carrier-added (n.c.a.) [^18^F]fluoride was produced via the ^18^O(p,n)^18^F nuclear reaction from [^18^O]H_2_O (Rotem Industries Ltd, Hyox oxygen-18 enriched water, min. 98%) on an ACSI TR19/9 Cyclotron (Advanced Cyclotron Systems Inc., Richmond, Canada). Cyclotron-produced [^18^F] fluoride was trapped on a Waters SepPak® light QMA anion exchange cartridge.

#### Radiosynthesis of [^18^F]fluoroazomycin arabinoside ([^18^F]FAZA), [^18^F]fluoro-misonidazole ([^18^F]FMISO) and 4-[^18^F]fluorobenzyl amine ([^18^F]FBA)

[^18^F] FAZA and [^18^F] FMISO were prepared according to literature procedures (Sorger et al. 2003; Hayashi et al. 2011; Kurihara et al. 2012) with some modifications in a GE TRACERlab™ FX automated synthesis unit. A solution of aminopolyether 2.2.2 (Kryptofix 222) (50 mg) and K_2_CO_3_ (1.8 mg) in CH_3_CH/water (86%, 1 mL) was used to elute the n.c.a. [^18^F]fluoride (16–22 GBq) from the QMA cartridge into a glassy carbon reactor. CH_3_CN (1.5 mL) was added to the reactor, and azeotropic drying was performed at 50 °C and 85 °C under a nitrogen flow under a vacuum for 15 min.

For the radiosynthesis of [^18^F]FAZA, this was followed by the addition of a solution of 1-α-D-(2,3-diacetyl-5-tosyloxy-arabinofuranosyl)-2-nitroimidazole (5 mg, 10.3 µmol) in dimethylsulfoxide (0.8 mL) and the mixture was heated for 10 min at 120 °C. After cooling at room temperature, 0.1 M NaOH (1.0 mL) was added for 3 min at room temperature to ensure complete deprotection. Finally, [^18^F]FAZA was neutralized with the addition of 0.2 M AcOH (1.5 mL) and the reaction mixture was passed through a Waters SepPak Alumina N Light cartridge into a sealed product vial.

The collected eluent (3.5 mL) was directly injected onto HPLC, using a Phenomenex Luna C18(2) column with an isocratic elution profile of 8% EtOH in water at flow rate of 2 ml/min. [^18^F]FAZA retention time under this conditions was 31–34 min.

For [^18^F]FMISO, a solution of 1- (2′-nitro-1′-imidazolyl) -2-*O*-tetrahydropyranyl-3-*O*-toluenesulfonylpropane-diol (NITTP, 5 mg) in CH_3_CH (1.0 mL) was added to the reactor and the mixture was heated at 100 °C for 10 min. After cooling at room temperature, 1 M HCl (1.0 mL) was added for 10 min at 100 °C to ensure complete deprotection. After cooling to room temperature, the reaction was neutralized with 30% w/v sodium acetate solution in water (1.0 mL). The reaction mixture was then passed through a Waters SepPak Alumina N Light cartridge into a sealed product vial. The collected eluent (3.0 mL) was directly injected onto HPLC, using a Phenomenex Luna C18(2) column with an isocratic elution profile of 8% EtOH in water at a flow rate of 2 ml/min. [^18^F]FMISO retention time under this conditions was 14.5 to 17.4 min.

[^18^F]FBA was synthesized according to the published automated synthesis procedure (Way and Wuest 2013).

#### Radiosynthesis of *N*-(4-[^18^F]fluorobenzyl)-2-(2-nitro-1*H*-imidazol-1-yl)acetamide ([^18^F]FBNA)

The radiosynthesis of [^18^F]FBNA was performed manually. Compound **3** (5 mg) and 1-ethyl-3-(3-dimethylaminopropyl)carbodiimide (EDC) (4.5 mg, (1 equiv.) dissolved in DMSO (500 µL) was reacted in a sealed vial with [^18^F]FBA (~ 740 MBq) in THF (500 µL), and 25 µL (2.6 equiv.) of DIPEA. The reaction was optimized by screening different temperatures (room temperature, 65 °C, 85 °C and 100 °C) in a heating block with agitation. Upon completion, the reaction was allowed to cool for 5 min prior to HPLC purification. The crude reaction mixture was diluted in Mili Q water (1 mL) and injected onto a HPLC column for purification.

HPLC purification was completed on the Gilson HPLC systems (HPLC Column is a Phenomenex LUNA® C18(2) column (100 Å, 250 × 10 mm, 10 µm) using gradient elution (A: water; B: CH_3_CN; 3 mL/min, 0 min 5% B, 3.5 min to 23 min gradient to 90% B, 25 min 90% B). The retention time of [^18^F]FBNA was 20–21 min as confirmed by the co-injection with reference compound. The collected HPLC fraction was concentrated, and the solvent was removed at 50 °C under reduced pressure. Purified radiotracer [^18^F]FBNA was then reconstituted in saline containing 8% of EtOH.

### Quality control

Aliquots of purified radiotracer [^18^F]FBNA and reference compound were analyzed by HPLC to determine chemical purity, radiochemical purity, and confirm chemical identity. HPLC analysis involved the following conditions: Stationary phase: Agilent C18, 4.6 mm × 250 mm, 5 μ, 100 Å; -Mobile phase: Solvent (A) Water Milli Q + 0.1% TFA, Solvent (B) 100% CH_3_CN; Isocratic elution: 70% (A), 30% B for 30 min; flow rate: 1 mL/min; Detection wave length: 280 and 300 nm. The pH of the final solution was determined using pH test strips; an aliquot of the sample was deposited in the test region of the indicator strip and a colorimetric comparison was made with the standard provided by the manufacturer. The chemical identity of radiotracer [^18^F]FBNA was determined by co-injection with the reference compound.

The molar activity of radiotracers [^18^F]FBNA, [^18^F]FAZA and [^18^F]FMISO was determined using HPLC analysis and calculated at the end of the radiosynthesis using a calibration curve (nmol of the respective reference compound *versus* area under the curve recorded in the analytical HPLC profile at 300 nm). To calculate the molar activity, the injected activity of the respective radiotracer was divided by the quantities in nmol of the reference compound that was extrapolated from the calibration curve.

### In vitro* stability studies*

The stability of radiotracer [^18^F]FBNA was studied at room temperature in physiological saline solution (0.9% NaCl) and pH = 7.0. The samples (~ 3.7 MBq) had a final volume of 500 µL. The stability of the compound was monitored via HPLC analysis over a time course of 6 h. The stability of radiotracer [^18^F]FBNA was also studied in mouse serum to assess its suitability in vivo experiments. 50 µL (3.7 MBq) aliquots of the radiotracer was added to 450 µL of freshly separated mouse serum, and the mixture was incubated at 37° C for up to 6 h. 20 µL aliquots were removed at different time points (0 h, 1 h, 3 h and 6 h). CH_3_CN (20 µL) was added for the precipitation of serum proteins. After centrifugation (5000× *g*, for 10 min), the supernatant was analyzed by HPLC as described in the previous section. All experiments were performed in triplicate.

### Determination of lipophilicity

The shake-flask method was used to determine the lipophilicity of [^18^F]FBNA (Wilson et al. 2001). The partition coefficient of [^18^F]FBNA was measured using *n*-octanol as the organic phase and PBS (pH 7.4) as the aqueous layer. Then, 500 µL of each layer was added to a LoBind Eppendorf tube, to which 50 µL (< 1 MBq) of [^18^F]FBNA was added, and the mixture was shaken vigorously for 5 min. The mixture was then centrifuged at 2000 rpm for 2 min to allow the layers to separate. Aliquots of 100 μL were removed from each layer, and the amount of [^18^F]FBNA present in each layer was measured in a Wizard gamma counter. Experiments were performed in triplicate.

### Cellular uptake studies

MKN45 cells (American Type Culture Collection, Manassas, VALLC (Tamura et al. 1996) and AGS cells (ATCC CRL-1739) were cultured in RPMI (Gibco, Life technologies, MD, USA). Both cell lines were supplemented with 10% fetal bovine serum (Gibco, Life technologies, MD, USA) and 50 µg/mL of gentamicin (Gibco, Life technologies, MD, USA). Mycoplasma contamination in cultured cells was excluded using Lonza Mycoplasma Detection Kit.

For cell uptake experiments, 5 × 10^5^ cells were seeded in two 6-well plates and grown for 24 h at 37 °C and 5% CO_2_ and 21% O_2_. After this period, one plate remained under normoxic conditions (21% of O_2_). The other plate was cultured in the H35 Hypoxystation (Don Whitley Scientific Ltd, Shipley) under hypoxic conditions (37 °C, 5% CO_2_ and 1% of O_2_) for 48 h. Cells were then incubated with 0.37 MBq of [^18^F]FBNA or [^18^F]FAZA (3 wells per plate) for 1 h at 37 °C. At the end of the experiment, cells were washed 5 times with ice-cold PBS and 1.0 M NaOH was added to lyse the cells and remove them into a gamma-counter tube. The radioactivity in each tube was counted in a Cobra II gamma counter (Packard, EUA). The experiments were done three times (n = 3) and GraphPad Prism 7 Software (San Diego, CA, USA) was used to analyze the data.

## Data Availability

The datasets used and/or analyzed during the current study are available from the corresponding author upon reason-able request.

## Abbreviations

[^18^F]FAZA: [^18^F]Fluoroazomycin Arabinoside
[^18^F]FBA: 4-[^18^F]fluorobenzylamine
[^18^F]FBN: 4-[^18^F]fluorobenzonitrile
[^18^F]FBNA: N-(4-[^18^F]fluorobenzyl)-2-(2-nitro-1H-imidazol-1-yl)acetamide
[^18^F]FETA: [^18^F]fluoroetanidazole
[^18^F]FETNIM: [^18^F]fluoroerythronitroimidazole
[^18^F]FMISO: [^18^F]Fluoromisonidazole
[^18^F]HX4: [^18^F]labeled flortanidazole
[^64^Cu]ATSM: [^64^Cu]Diacetyl-bis(N4-methylthiosemicarbazone)
FBNA: *N*-(4-Fluorobenzyl)-2-(2-nitro-1H-imidazol-1-yl) acetamide
HIF-1: Hypoxia-inducible factor 1
PET: Positron Emission Tomography
Rmax: Maximum Ratio
SUVmax: Maximum Standardized Uptake Value
